# Validation of a commercially available CAD-system for lung nodule detection and characterization using CT-scans

**DOI:** 10.1007/s00330-024-10969-0

**Published:** 2024-07-23

**Authors:** Jasika Paramasamy, Souvik Mandal, Maurits Blomjous, Ties Mulders, Daniel Bos, Joachim G. J. V. Aerts, Prakash Vanapalli, Vikash Challa, Saigopal Sathyamurthy, Ranjana Devi, Ritvik Jain, Jacob J. Visser

**Affiliations:** 1 Department of Radiology and Nuclear Medicine, Erasmus Medical Center, Dr. Molewaterplein 40, 3015 GD Rotterdam, The Netherlands; 2 Qure.ai, Level 7, Oberoi Commerz II, Goregaon East, Mumbai, 400063 India

**Keywords:** Lung, Lung cancer, Validation study, Early detection of cancer, Computer-assisted detection

## Abstract

**Objectives:**

This study aims to externally validate a commercially available Computer-Aided Detection (CAD)-system for the automatic detection and characterization of solid, part-solid, and ground-glass lung nodules (LN) on CT scans.

**Methods:**

This retrospective study encompasses 263 chest CT scans performed between January 2020 and December 2021 at a Dutch university hospital. All scans were read by a radiologist (R1) and compared with the initial radiology report. Conflicting scans were assessed by an adjudicating radiologist (R2). All scans were also processed by CAD. The standalone performance of CAD in terms of sensitivity and false-positive (FP)-rate for detection was calculated together with the sensitivity for characterization, including texture, calcification, speculation, and location. The R1’s detection sensitivity was also assessed.

**Results:**

A total of 183 true nodules were identified in 121 nodule-containing scans (142 non-nodule-containing scans), of which R1 identified 165/183 (90.2%). CAD detected 149 nodules, of which 12 were not identified by R1, achieving a sensitivity of 149/183 (81.4%) with an FP-rate of 49/121 (0.405). CAD’s detection sensitivity for solid, part-solid, and ground-glass LNs was 82/94 (87.2%), 42/47 (89.4%), and 25/42 (59.5%), respectively. The classification accuracy for solid, part-solid, and ground-glass LNs was 81/82 (98.8%), 16/42 (38.1%), and 18/25 (72.0%), respectively. Additionally, CAD demonstrated overall classification accuracies of 137/149 (91.9%), 123/149 (82.6%), and 141/149 (94.6%) for calcification, spiculation, and location, respectively.

**Conclusions:**

Although the overall detection rate of this system slightly lags behind that of a radiologist, CAD is capable of detecting different LNs and thereby has the potential to enhance a reader’s detection rate. While promising characterization performances are obtained, the tool’s performance in terms of texture classification remains a subject of concern.

**Clinical relevance statement:**

Numerous lung nodule computer-aided detection-systems are commercially available, with some of them solely being externally validated based on their detection performance on solid nodules. We encourage researchers to assess performances by incorporating all relevant characteristics, including part-solid and ground-glass nodules.

**Key Points:**

- *Few computer-aided detection (CAD) systems are externally validated for automatic detection and characterization of lung nodules.*
- *A detection sensitivity of 81.4% and an overall texture classification sensitivity of 77.2% were measured utilizing CAD.*
- *CAD has the potential to increase single reader detection rate, however, improvement in texture classification is required.*

**Graphical Abstract:**

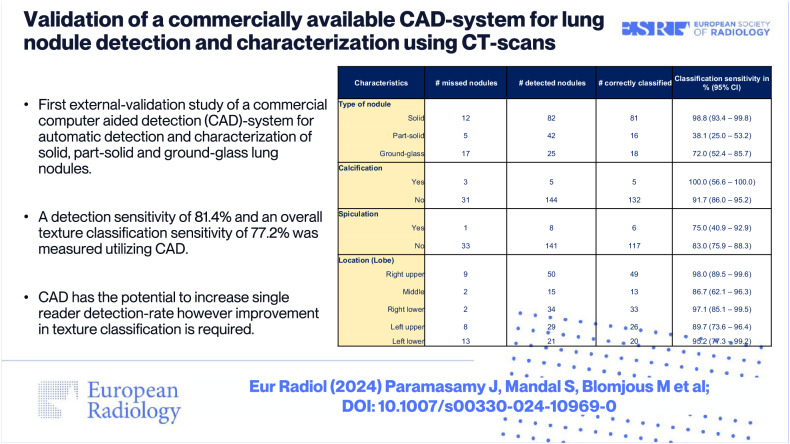

## Background

Lung cancer is the leading cause of cancer mortality worldwide that claimed approximately 2 million lives in 2020 [1]. Despite the poor prognosis of lung cancer, early detection of malignant lung nodules (LNs) has been shown to substantially improve patient survival as these lesions are mostly amenable to curative interventions. Incidental LNs are reported to be present in 30-50% of all chest Computed Tomography (CT)-scans [2–4], making them a crucial resource for LN detection.

Due to the shortage of radiologists and increasing workload [5], actionable nodules might be overlooked during the radiological interpretation. Previous studies demonstrated an LN-detection sensitivity ranging between 65 and 85% [6–8] in individual readers, depending on the indication, nodule size, the expertise of the readers, and time allowed for assessment. Double reading would improve the detection rate to 70–95% [7, 9, 10]. However, in routine clinical practice, double reading is not customary, as it would be tedious and time-consuming.

Acknowledging this challenge, software companies have progressively delved into the development of Computer-Aided Detection (CAD)-systems as second reader, with some of them already commercially accessible. Promising diagnostic performances have been reported by vendors based on internal study results. While internal validation is a mandatory step in the field of software development, external validation provides significant added value by evaluating a CAD system’s performance using new, independent datasets. Regrettably, solely 6% of all developed algorithms are externally validated [11, 12].

Besides the lack of external validation, numerous vendors prioritize detection of LNs over automated capturing all clinically relevant characteristics such as calcification, spiculation and location. Moreover, texture type is one of the important characteristics in determining the malignancy risk of a nodule [13–16]. In fact, it is well demonstrated that a majority of persisting subsolid nodules (encompassing both ground-glass and part-solid nodules) represent lung adenocarcinomas across diverse phases [17].

To the best of our knowledge, there are only a few commercially available tools capable of assessing characterizations and only a small subset among them can differentiate between all three texture types. The characterization sensitivity of all these commercial algorithms is not externally validated nor publicly available.

In this study, the performance of a commercially available artificial intelligence (AI)-based CAD system, which asserts automatic detection and accurate classification of solid, part-solid, and ground-glass nodules in CT scans, is externally validated.

## Methods

The institutional review board waived the requirement for informed patient consent for this retrospective study.

### Study population

According to our sample size calculation (see Supplementary Material– Sample Size), our study required approximately 110 nodule-containing scans, alongside a similar number of non-nodule containing scans.

Chest CT scans taken between January 2020 and December 2021 at Erasmus Medical Center, a Dutch University Hospital were retrospectively evaluated. We performed a systematic query in our Picture Archiving and Communicating System (PACS) on chest CT scans of subjects aged ≥ 18 years, using the following keywords: “solid nodules”, “ground-glass nodule”, “mixed nodule” and “part-solid nodule” and their inflections. Among these, we selected scans aiming to realize the stratification criteria (see Supplementary Material- Table S1) to ensure all texture types were adequately represented. Exclusions encompassed scans with metal artifacts, excessive motion artifacts, disordered slices, > 10 nodules, slice-thickness > 5 mm, and absent radiologist reports. Scans were screened and included till the aforementioned stratification criteria were satisfied.

For our study, 263 consecutive scans were included based on the radiology report: 113 nodule-containing scans and 150 non-nodule-containing scans. Note that the final number may change after ground truthing (GT).

### Reference standard

A pulmonary nodule was defined as a lesion sized 3–30 mm in diameter as indicated in the Fleischner glossary [15, 18, 19].

The reference standard was based on the expertise of three radiologists (all with at least eight years of experience), these are the initial radiology reporter, annotator (R1), and arbitrator (R2).

As mentioned earlier, CT examinations were included based on the initial radiology reports, from which nodule information was extracted. Thereafter, all included CT examinations were read by a radiologist (R1) using an advanced annotation platform RedBrick.AI (Claymonth, Delaware, USA). Blinded to the original report, R1 annotated nodules per slice and specified texture, location, and the presence of calcification or spiculation. The average diameter and volume of each nodule were calculated.

All annotations made by R1 were compared with the original radiology report. When a nodule was identified by R1 but not mentioned in the initial radiology report, or vice versa, it was considered to be a discrepant interpretation. These discrepancies were reviewed by a second radiologist (R2), who had access to both annotations. R2 was tasked with arbitrating between the original radiology report and R1’s findings.

All board-certified readers were blinded to CAD findings.

### Artificial intelligence based CAD system

All scans were processed by qCT v1.1 (Qure.ai, Mumbai, India), a commercially available AI-based medical device [20]. This algorithm detects pulmonary nodules and provides information on their location, texture, calcification/spiculation status, as well as the average diameter/volume.

During internal validation, qCT demonstrated an 82% nodule-level detection sensitivity. Characterization sensitivities were 82% for texture, 91% for calcification, 82% for spiculation, and 96% for location.

More information about the qCT software can be found in the Supplementary Materials- Specifications CAD.

### Statistical analysis

#### Descriptive analysis

After ground-truthing, scans were categorized into a nodule-containing and non-nodule-containing scan group. We compared demographic (age, sex), clinical (presence/absence of other chest abnormalities such as atelectasis, fibrosis, pericardial fluid etc.), and acquisition parameters (machine-type, scan-type) between these groups. Age was dichotomized at 55, considering prior research indicating increased LN occurrence above this age [21].

Variables for nodule-containing and non-nodule-containing scans were summarized using numbers and percentages. We used Chi-square tests to assess the differences between these scans. Throughout the entire study, we utilized a *p*-value threshold of ≤ 0.05 to determine the statistical significance of the results.

#### Detection analysis

Analysis was conducted at both scan- and nodule-level, with a focus on nodule-level. CAD findings were scored using the 3D-intersection-over-union (IoU) method.

At the scan level, a scan was classified as true positive (TP) if CAD detected at least one nodule with a volume overlap of at least 10% with a GT nodule [22]. If the overlap was less than 10% or if the CAD failed to detect nodules in the nodule-containing scans, it was considered a false negative (FN) scan. A scan was labelled false positive (FP) if CAD detected anything in the non-nodule-containing scans and true negative if it detected nothing in the non-nodule-containing scans [23, 24]. The modified Wilson score method was used to construct the 95% confidence interval (CI) of sensitivity, specificity and precision. False positive per image (FPPI) at scan level was calculated by dividing the number of FP scans by the number of nodule-containing scans. Empirical Area Under the Receiver Operating Characteristic (AUC-ROC) and Precision-Recall (AUC-PR) curve analyses, along with the F1-score, were employed to assess CAD’s overall performance. DeLong’s method was utilized to construct the 95%CI for AUC-ROC, while the Clopper-Pearson method was used for the F1-score’s 95%CI. Note that recall is the same as sensitivity.

At the nodule level each CAD finding was assessed individually using the 10%-IoU-criteria. A CAD finding was considered TP if at least 10% of its volume had overlap with the GT nodule; FN if CAD did not detect the nodule or if the overlap was < 10%, and FP in case of a finding within the non-nodule-containing scan group. Here, sensitivity was calculated, and 95%CI was constructed using the method described by Rao and Scott for correlated data [25]. To demonstrate the effectiveness of the CAD system on CT scans, the performance was evaluated using the Free-response Receiver Operating Characteristic (FROC)-curve.

Nodule-level sensitivity across subgroups was also reported, as well as the R1 sensitivity.

#### Characterization analysis

The CAD system characterises nodules, which is summarised with numbers and percentages for detected and missed nodules. To evaluate the accuracy of the CAD system for characterizing detected nodules, the sensitivity (along with 95% Wilson Score CI) was calculated.

The absolute errors between GT and predicted diameters and volumes were calculated and summarized using the mean. Bland-Altman plots were presented to visualize the agreement between GT and predicted quantifications.

Statistical analyses were conducted using R v4.1.2 (R Core Team, 2021) in RStudio v2022.12.0 + 353 (R Studio Team, 2021). An overview of all the used packages can be found in the section Statistical Analysis Packages of the Supplementary Material.

## Results

**Fig. 1:**
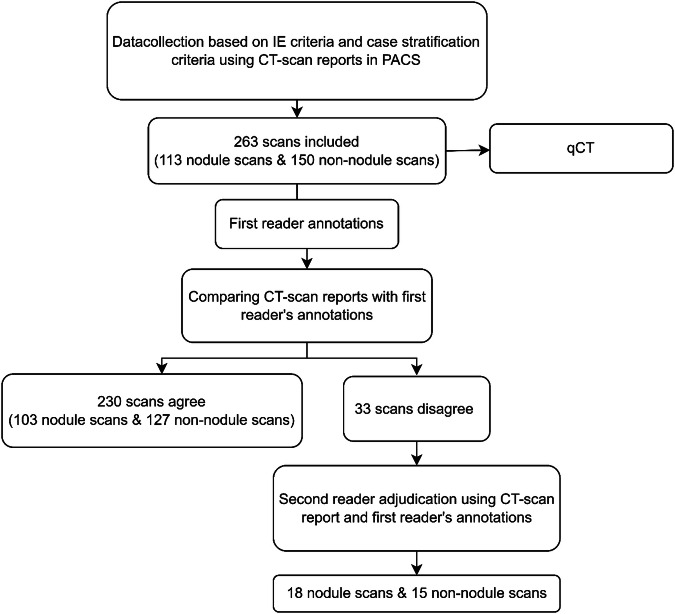
Flowchart of the workflow: All CT scan reports of scans acquired between January 2020 and December 2021 were screened, based on the inclusion and exclusion criteria and case stratification. All scans were included till satisfactory of the case stratification as shown in Table S1 of the Supplementary Material. A total of 263 scans were included, with 150 scans reporting no nodules and 113 scans mentioning nodules. Thereafter the included scans were processed by the AI-based CAD and read by the first reader. The first reader was blinded to the report and was tasked to annotate nodules and assign their characteristics including texture, presence of calcification/spiculation and location. These annotations were compared with the radiology reports. In 230 scans the annotations reconciled the radiology reports while 33 cases exhibited discrepancies. A second radiologist reviewed all 33 cases using the radiology report and the first reader’s annotations. Eighteen of the disagreement scans turn out to be nodule-containing scans while 15 are non-nodule-containing scans. Neither reader had access to the CAD’s output during this process

Based on the radiology report, a total of 263 CT scans were included. After the first reading process, 230 were in agreement with the original report. For all 33 disagreement scans, R2 adjudicated. The overall workflow is shown in Fig. 1.

Out of the 263 scans, 121 exhibited at least one nodule, while the remaining 142 did not contain any nodules. Table 1 shows the demographic, clinical and CT acquisition parameters in nodule-containing and non-nodule-containing scans. As anticipated, within the group of patients aged < 55 years, the percentage of nodule-containing scans were lower compared to patients aged ≥ 55 years (23.5% vs 60.2%). Furthermore, no significant differences were observed in gender, machine type, and scan type between the nodule- and non-nodule-containing scan group (*p* = 0.5, *p* = 0.3 and *p* = 0.2, respectively).

**Table 1:** Clinical, demographic, and CT acquisition variables in nodule (*n* = 121) and non-nodule scans (*n* = 142)

Variables^a^	Number of nodule scans *n* = 121 (proportion in %)	Number of no-nodule scans *n* = 142 (proportion in %)	*p*-value
**Age**			
< 55 years	24 (23.5)	78 (76.5)	< 0.001
≥ 55 years	97 (60.2)	64 (39.8)	
**Sex**			
Male	63 (48.5)	67 (51.5)	0.5
Female	58 (43.6)	75 (56.4)	
**Other abnormality**			
Yes	66 (52.8)	59 (47.2)	0.05
No	55 (39.9)	83 (60.1)	
**Machine type**			
SOMATOM Edge Plus	42 (46.2)	49 (53.8)	0.3
SOMATOM Definition Edge	27 (44.3)	34 (55.7)	
SOMATOM Force	34 (54.8)	28 (45.2)	
SOMATOM Drive	15 (34.1)	29 (65.9)	
Others^b^	3 (60.0)	2 (40.0)	
**Scan type**			
Noncontrast	71 (42.5)	96 (57.5)	0.2
Contrast	50 (52.1)	46 (47.9)	

^a^ All scans had a slice-thickness of 1 mm

^b^ Others includes Biograph128, and NAEOTOM Alpha

### Scan-level

**Fig. 2:**
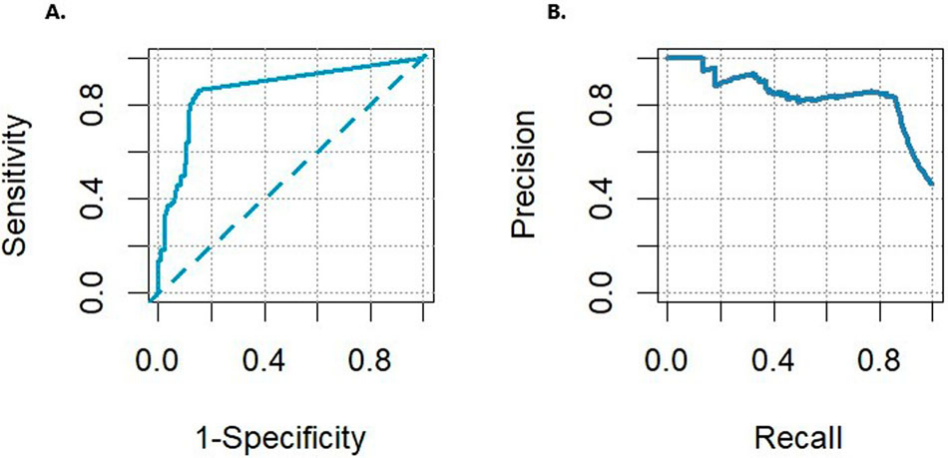
**A** Receiver operating characteristics curve and (**B**) Precision recall curve**:** on the left (**A**), the ROC curve of the CAD system is depicted, the AUC-ROC is equal to 0.865 (CI:0.837–0.892). On the right (**B**), the PR curve of the concerned system is shown, the AUC-PR is equal to 0.844. Note that sensitivity is also known as recall

Out of the 121 nodule scans, 104 were correctly flagged as having nodules; of the 142 non-nodule-containing, 121 scans were correctly not flagged for nodules. CAD demonstrated a scan-level sensitivity of 104/121 (86.0%; CI: 78.6%–91.0%), specificity of 121/142 (85.2%; CI: 78.4%–90.1%) and precision of 104/125 (83.2%; CI: 75.7–88.7%). The AUC-ROC (Fig. 2A) was 0.865 (CI:0.837–0.892), AUC-PR (Fig. 2B) was 0.844 and F1-score was 0.846 (CI: 0.794–0.888), resulting in an overall FPPI of 21/121 (0.174) per scan.

### Nodule-level (detection)

**Fig. 3:**
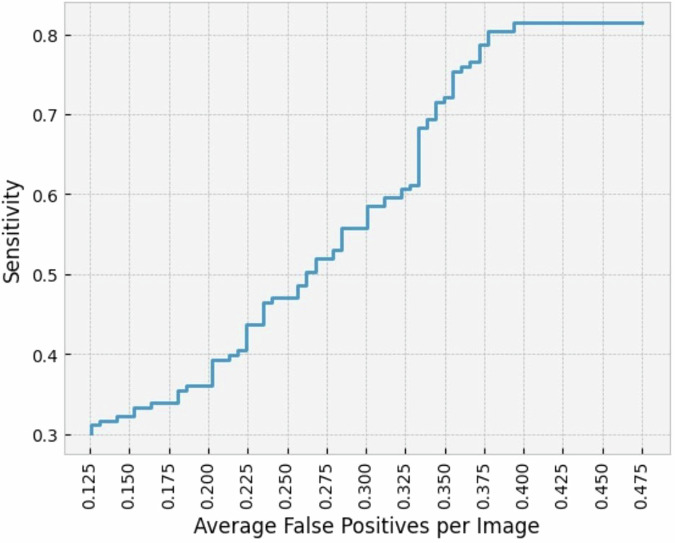
Free response operating characteristic plot: Average false positive rate on the x-axis and the corresponding sensitivity on the y-axis

One hundred and eighy-three nodules were identified on 121 nodule scans according to the reference standard, yielding an average of 1.51 nodules per nodule-containing scan. In these patients, the CAD system detected 149 nodules with a mean IoU of 0.417 (CI: 0.396–0.438) and missed 34 nodules. Forty-nine FP findings were obtained within the nodule scans, whereas 23 FP findings were observed within the non-nodule-containing scans. The CAD detection system had a nodule-level sensitivity of 149/183 (81.4%; CI: 76.2%–86.6%) with an average of 49/121 (0.405) FPs per nodule-containing examination. The FROC plot in Fig. 3 shows the sensitivity of the CAD-system at various FP-rates. The sensitivity by subgroups is reported in Table 2. The nodule-level sensitivity does not appear to change considerably among the subgroups. However, within the machine type subgroup an outlier in the group “Others” is observed with a 50.0% sensitivity, nevertheless this discrepancy might be attributed to the small sample size of 4.

**Table 2:** Subgroup nodule-level detection sensitivity of the total 183 GT nodules

Variable^a^	Sensitivity in % (95%CI)
**Age**	
< 55 years (*n* = 31)	77.4 (63.2–91.7)
≥ 55 years (*n* = 152)	82.2 (76.7–87.8)
**Sex**	
Male (*n* = 95)	82.1 (75.0–89.2)
Female (*n* = 88)	80.7 (73.0–88.3)
**Other abnormalities**	
Yes (*n* = 98)	81.6 (74.8–88.5)
No (*n* = 85)	81.2 (73.3–89.1)
**Machine type**	
SOMATOM Edge Plus (*n* = 65)	78.5 (68.8–88.1)
SOMATOM Definition Edge (*n* = 42)	78.6 (67.4–89.7)
SOMATOM Force (*n* = 51)	86.3 (78.0–94.6)
SOMATOM Drive (*n* = 21)	90.5 (77.8–100.0)
Others^b^ (*n* = 4)	50.0 (7.6–92.4)
**Scan type**	
Noncontrast (*n* = 110)	79.1 (72.6–85.6)
Contrast (*n* = 73)	84.9 (76.6–93.2)

^a^ All scans had a slice-thickness of 1 mm

^b^ Others includes Biograph128 and NAEOTOM Alpha

The detection sensitivity per texture type was 82/94 (87.2%), 42/47 (89.4%) and 25/42 (59.5%) for solid, part-solid and ground-glass, respectively (Table 3).

**Table 3:** GT-nodule characteristics among CAD detected (*n* = 149) and missed nodules (*n* = 34)

Characteristics	Number of detected nodules (proportion in %)	Number of missed nodules (proportion in %)
**Type of nodule**		
Solid	82 (55.0)	12 (35.3)
Part-solid	42 (28.2)	5 (14.7)
Ground-glass	25 (16.8)	17 (50.0)
**Calcification**		
Yes	5 (3.3)	3 (8.8)
No	144 (96.7)	31 (91.2)
**Spiculation**		
Yes	8 (5.4)	1 (2.9)
No	141 (94.6)	33 (97.1)
**Location (Lobe)**		
Right upper	50 (33.6)	9 (26.5)
Middle	15 (10.1)	2 (5.9)
Right lower	34 (22.8)	2 (5.9)
Left upper	29 (19.5)	8 (23.5)
Left lower	21 (14.1)	13 (38.4)
**Diameter**^a^		
< 6 mm	78 (52.3)	21 (61.8)
6–8 mm	29 (19.5)	8 (23.5)
> 8 mm	42 (28.2)	5 (14.7)
**Volume**		
< 100 mm^3^	33 (22.1)	12 (35.3)
100–250 mm^3^	35 (23.5)	11 (32.4)
> 250 mm^3^	81 (54.4)	11 (32.4)

^a^ dimensions are the average of long and short axes diameters

### Nodule-level (characterization)

In order to evaluate the accuracy of the CAD system in characterizing each nodule, we only focused on those true nodules. Table 3 displays the distribution of GT-nodule characteristics among CAD detected and missed nodules. A total of 149 nodules were detected and 34 nodules were missed. Remarkably, half of the missed nodules (17) are ground-glass nodules. The distribution of calcification and spiculation is comparable between detected and missed nodules. Furthermore, it is noteworthy that a substantial portion of the missed nodules is localized in the left lower lobe (38.4%).

Characterization sensitivity of CAD was computed for the correctly detected nodules (Table 4). The overall accuracy was: texture 116/149 (77.2%; CI: 69.8–83.2%), calcification 137/149 (91.9%; CI: 86.5–95.3%), spiculation 123/149 (82.6%; CI: 75.7–87.8%), and location 141/149 (94.6%; CI: 89.8–97.3%).

**Table 4:** Characterization accuracy of the detected nodules by the CAD-system (*n* = 149)

Characteristics	Number of correctly classified	Proportion correctly classified = sensitivity in % (95%CI)
**Type of nodules**		
Solid (*n* = 82)	81	98.8 (93.4–99.8)
Part-solid (*n* = 42)	16	38.1 (25.0–53.2)
Ground-glass (*n* = 25)	18	72.0 (52.4–85.7)
**Calcification**		
Yes (*n* = 5)	5	100.0 (56.6–100.0)
No (*n* = 144)	132	91.7 (86.0–95.2)
**Spiculation**		
Yes (*n* = 8)	6	75.0 (40.9–92.9)
No (*n* = 141)	117	83.0 (75.9–88.3)
**Location (Lobe)**		
Right upper (*n* = 50)	49	98.0 (89.5–99.6)
Middle (*n* = 15)	13	86.7 (62.1–96.3)
Right lower (*n* = 34)	33	97.1 (85.1–99.5)
Left upper (*n* = 29)	26	89.7 (73.6–96.4)
Left lower (*n* = 21)	20	95.2 (77.3–99.2)

The overall mean absolute error in diameter was 0.57 mm (CI: 0.38 mm–0.76 mm). When stratified by diameter categories of < 6 mm, 6–8 mm, and > 8 mm, the errors were 0.37 mm (CI: 0.23 mm–0.52 mm), 0.56 mm (CI: 0.23 mm–0.89 mm), and 0.96 mm (CI: 0.39 mm–1.53 mm), respectively.

For volume, the overall mean absolute error was 147.14 mm^3^ (CI: 97.97 mm^3^–206.31 mm^3^). Stratified by volume categories of < 100 mm^3^, 100–250 mm^3^, and > 250 mm^3^, the errors were 16.97 mm^3^ (CI: 9.15 mm^3^–24.79 mm^3^), 32.60 mm^3^ (CI: 21.92 mm^3^–43.28 mm^3^), and 249.67 mm^3^ (CI: 145.87 mm^3^–353.47 mm^3^), respectively.

**Fig. 4:**
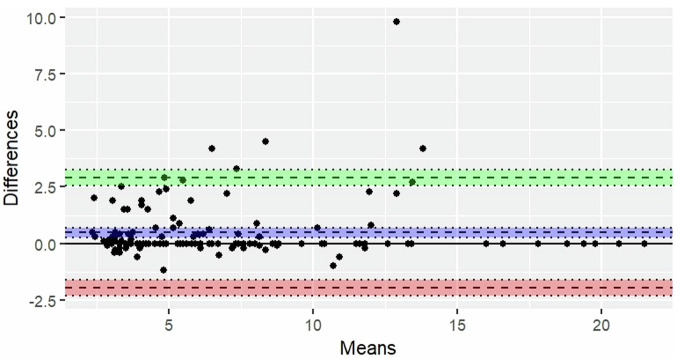
Bland-Altman plot for diameter: The Bland-Altman plot illustrates the agreement between the ground truth and predicted diameter. The mean of the differences (bias) and the limits of agreement (LoA), along with their 95%CI, are depicted. The mean difference between the two values is 0.47 mm (CI: 0.27 mm–0.68 mm). The bias is represented by a blue area, with a dashed line indicating the point estimate of the bias and a dotted line representing the corresponding 95%CI. The upper LoA is 2.90 mm (CI: 2.56 mm–3.25 mm). The upper LoA is illustrated by a green area, with a dashed line indicating the point estimate and a dotted line representing the 95%CI. The lower LoA is -1.95 mm (CI: -2.30 mm to -1.61 mm). The lower LoA is depicted by a red area, with a dashed line indicating the point estimate and a dotted line representing the 95%CI

**Fig. 5:**
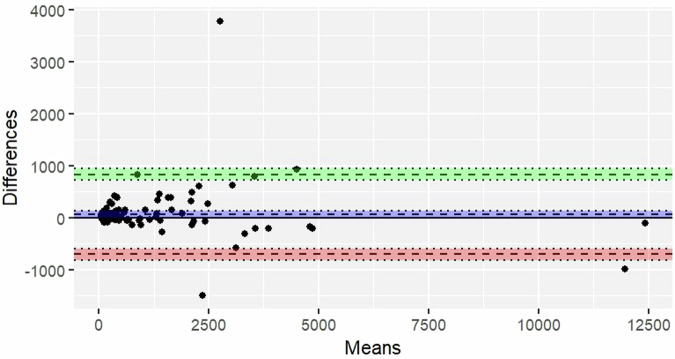
Bland-Altman plot for volume: The Bland-Altman plot illustrates the agreement between the ground truth and predicted volume. The mean of the differences (bias) and the limits of agreement (LoA), along with their 95% confidence intervals (CI), are depicted. The mean difference between the two values is 61.8 mm^3^ (CI: -1.68 mm^3^–125 mm^3^). The bias is represented by a blue area, with a dashed line indicating the point estimate of the bias and a dotted line representing the corresponding 95%CI. The upper LoA is 830 mm^3^ (CI: 722 mm^3^–939 mm^3^). The upper LoA is illustrated by a green area, with a dashed line indicating the point estimate and a dotted line representing the 95%CI. The lower LoA is -707 mm^3^ (CI: -815 mm^3^ to -598 mm^3^). The lower LoA is depicted by a red area, with a dashed line indicating the point estimate and a dotted line representing the 95%CI

The bias and limits of agreement for diameter and volume are reported in Fig. 4 and Fig. 5, respectively.

### Analysis conflicting scans

In the 33 conflicting scans (18 nodule-containing and 15 non-nodule-containing scans), R1’s interpretations did not align with the radiology reports. Following adjudication, 27 true nodules were identified within these scans. The CAD-system detected 19 true nodules and 10 false nodules. R1 identified 9 true nodules but also marked 39 FPs, however it is important to note that these 39 findings are related to the total dataset of 263 scans. Table 5 summarizes characteristics of the 27 true nodules within the disagreement scans and provides an overview of the nodules found and/or missed by R1 and CAD. A total of 165 true nodules were identified by R1, yielding a sensitivity of 165/183 (90.2%).

**Table 5:** GT characteristics of the total number of lung nodules (*n* = 27), lung nodules only identified by R1 (*n* = 2), lung nodules only detected by CAD (*n* = 12), mutually found lung nodules by R1 and CAD (*n* = 7) and lung nodules not found by R1 nor CAD (*n* = 6) within the disagreement scans

	GT identified nodules	Only R1 identified nodules	Only CAD detected nodules	R1 and CAD mutually found nodules	Not found by both
**Number of nodules**	27	2	12	7	6
**Type of nodule**					
Solid	20	2	8	6	4
Part-solid	3	0	2	1	0
Ground-glass	4	0	2	0	2
**Calcification**					
Yes	1	0	0	0	1
No	26	2	12	7	5
**Spiculation**					
Yes	2	0	1	1	0
No	25	2	11	6	6
**Location (Lobe)**					
Right upper	3	0	0	1	2
Middle	5	0	3	2	0
Right lower	9	0	6	3	0
Left upper	3	0	0	1	2
Left lower	7	2	3	0	2
**Diameter**					
< 6 mm	23	2	9	6	6
6–8 mm	2	0	1	1	0
> 8 mm	2	0	2	0	0
**Volume**					
< 100 mm^3^	13	2	5	4	2
100–250 mm^3^	10	0	5	1	4
> 250 mm^3^	4	0	2	2	0

## Discussion

This external validation study evaluated the standalone performance of a commercial AI-based CAD system designed to automatically detect and characterize solid, part-solid, and ground-glass LNs in CT scans.

Based on the results of the reference standard, a total of 183 true nodules were identified. CAD successfully detected 149 nodules, achieving a detection sensitivity of 81.4%, which is slightly lower than R1’s detection performance (165 nodules with a sensitivity of 90.2%). Two out of 27 nodules within the discrepant scan findings were found by R1 but were missed by the CAD system, and 12 nodules were only detected by the CAD system. Although CAD detects not more nodules than R1, the system has the potential to increase the sensitivity of R1 by at least 7% (12/165) when used as the second reader. We believe that the primary benefit of deploying CAD lies in detecting LNs in the lower lobe regions as well as small nodules.

**Fig. 6:**
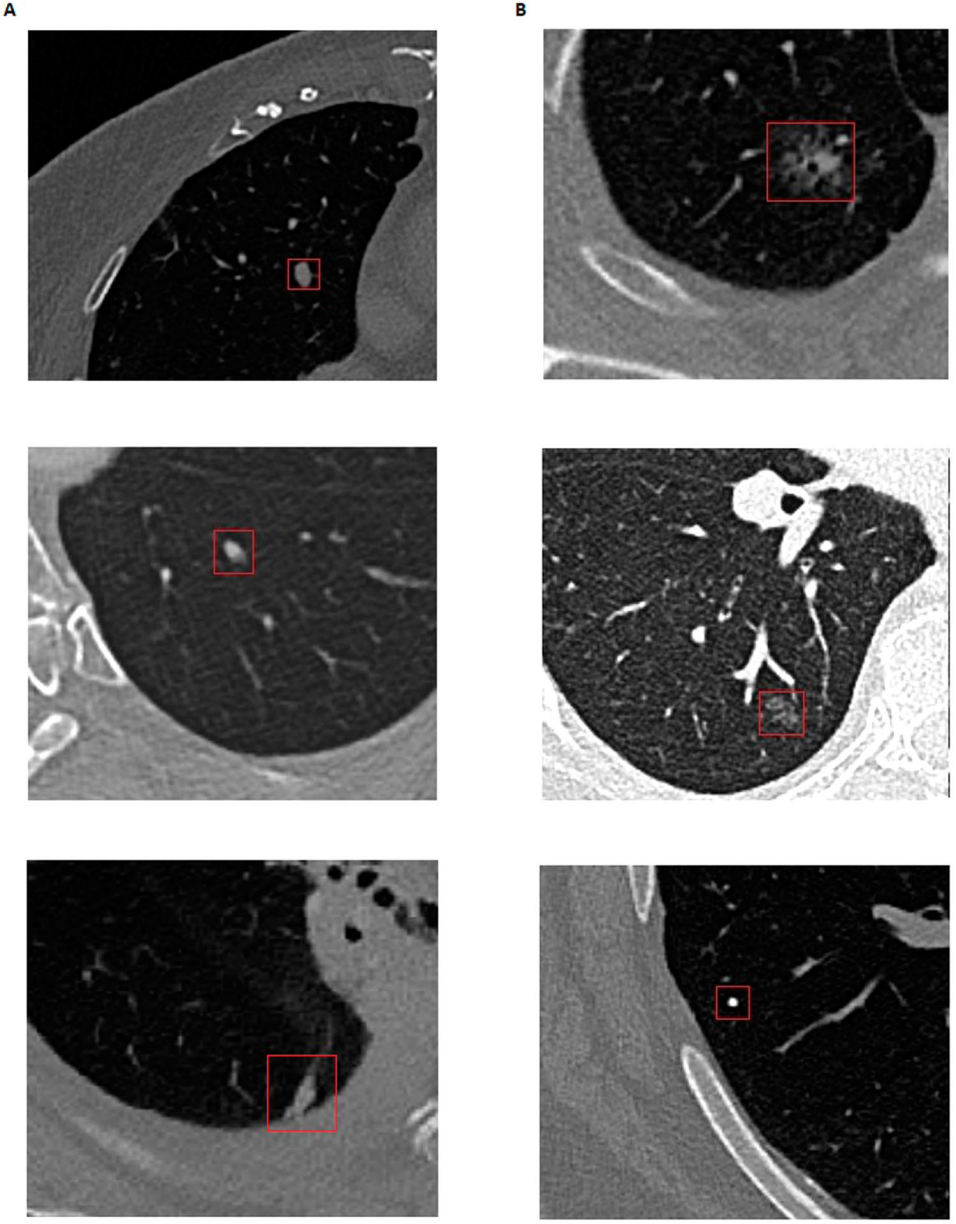
Examples of FP and FN findings: On the left (**A**), three examples of false positives (FP) are depicted. According to our reviewing radiologists, the top one represents an obvious blood vessel in the middle lobe; the middle CT scan contains slight motion artefacts, likely resulting in the incorrect flagging of a blood vessel in the left lower lobe; the bottom one is a perifissural lymph node. On the right (**B**), three false negatives (FN) are shown, these were missed by the CAD-system. The top image shows a part-solid nodule in the right lower lobe; the middle image displays a ground-glass nodule, also located in the right lower lobe; the bottom image features a subtle, calcified solid nodule situated in the right lower lobe

In the assessment of CAD’s performance, 72 findings were labelled as FPs and 34 findings as FNs. Nodule detection is an exceedingly intricate process, primarily owing to the complexity of nodules themselves, which in turn results in a notable incidence of FPs and FNs. FPs may arise from intrapulmonary lymph nodes and granulomas, while FNs can result from nodules situated adjacent to the pleural fissure or subtle nodules (e.g., small nodules or GGNs) that evade detection. Moreover, bronchovascular structures and technical artefacts further compound the risk of both FPs and FNs. Figure 6 illustrates several examples of FP and FN findings within our study.

The overall detection sensitivity achieved by the proposed CAD system is comparable to or slightly higher than the nodule-level sensitivity observed in other commercially available CAD algorithms. Lo et al (2018), for example, evaluated a commercial CAD system using scans from the National Lung Screening Trial. In this study, a nodule-level sensitivity of 82% was achieved at 0.75 FPPI [26]. The performance of another commercial CAD system was assessed in the study of Murchison et al (2022) using data from the United Kingdom. In this study, a sensitivity of 82.3% was reported at 1 FPPI, reaching a maximum sensitivity of 95.9% at an average FP-rate of 10.9 [27].

Besides detection performance, the characterization sensitivity of the system was also assessed in our study. Traditionally characterization relies on the subjective judgments of the evaluating radiologist, a process susceptible to variations from person-to-person [28–32]. This evaluation is crucial in determining the subsequent steps for patient care [13, 15, 18, 19]. Characterization using CAD can help to achieve a more objective approach for nodule assessment. In our study, an overall mean absolute error of 0.57 mm and 147.1 mm^3^ for diameter and volume, respectively, was measured between the GT nodule and CAD predicted nodule, which is considered acceptable and comparable with inter-reader variability [28–32]. The overall sensitivity for the other characterizations exceeded 80% (91.9% for calcification; 82.6% for spiculation and 94.6% for location). However, the accuracy for texture fell slightly below (77.2%), especially when considering ground-glass and part-solid nodules, current performance stands at 72.0% and 38.1%, respectively, indicating notable room for improvement. The technical challenge posed by both texture types is twofold: first, they need to be detected which is a significant difficulty in itself. Our study revealed 17/34 (50%) of missed nodules were ground-glass nodules (Table 3). Once detection is achieved, the subsequent step involves characterization. Given the technical challenge of detecting and distinguishing the textures, numerous AI-based CADs lack this essential functionality of characterization.

To date, this is the first study utilizing a commercially available CAD system capable of automatically detecting and characterizing all three LN texture types. Also, almost all studies primarily emphasize detection performance rather than characterization. Hence no comparison with other commercial tools is feasible. Nonetheless, there is one study which validated the characterization performance of another commercial CAD, however this system can solely differentiate between solid and subsolid nodules. The study reported a classification sensitivity of 98.8% for solid nodules and 68.4% for subsolid nodules [27].

One limitation of our study is that re-evaluation by the adjudicator was limited to the 33 conflicting scans. Therefore, one may question whether all 72 FP findings of CAD are correctly classified, since theoretically some may have been overlooked by R1 and the initial radiology report. Another drawback is that we did not provide precise data on the operation and computation time of the CAD system due to the absence of real-time clinical scenarios and the retrospective nature of our study. Furthermore, the exclusion of CT examinations containing artefacts and/or > 10 LNs make the selected scans not fully representative of real-world clinical scenarios. Nevertheless, it’s important to acknowledge that artefacts in scans also pose challenges for radiologists. Additionally, in practice, radiologists typically assess a maximum of 5–10 nodules per scan [33]. Despite these limitations, the main scope of our study was to determine the standalone performance of CAD using external data rather than to determine its impact in a real-world setting.

While the CAD tool shows comparable nodule detection to a single read, drawbacks, including a high FP-rate and the inability for rational decision-making based on the characterization and clinical context, highlight the preference of experienced radiologists to review scans independently. Increased FP-rate and inadequate classifications demands additional effort for radiologists to re-evaluate scans. Moreover, a significant portion of detected lesions proves to be benign upon further examination, emphasizing the essential role of radiologists in decision-making for follow-up procedures [34, 35].

Future research could compare radiologists’ performance in detecting and characterizing LNs with and without CAD-system support. In this way, we can assess whether radiologists’ LN detection-rates exhibit improvement and whether the characterization of nodules can be assessed more objectively when aided by an algorithm. Additionally, this investigation could elucidate the broader implications of integrating CAD into clinical practice, encompassing its effects on workflow optimization, diagnostic precision, and treatment decisions [36]. Additionally, exploration of cost-effectiveness, employing appropriate study designs, is imperative to demonstrate its economic viability within healthcare systems.

## Conclusion

In conclusion, the proposed commercially available CAD system exhibited detection performance slightly below that of a single reader but marginally better than or comparable to other commercially available LN detection applications. CAD detects different LNs compared to a single reader, indicating its potential to enhance a radiologist’s performance when employed as a second reader. While CAD shows promising characterization performance in identifying nodule size, location, spiculation status and calcification status, further refinements in texture classification are required.

## Supplementary information

Supplementary Material

## Abbreviations

AI: Artificial intelligence
AUC-ROC: Area under the receiver operating characteristic curve
AUC-PR: Area under the precision-recall curve
CAD: Computer-aided detection
CI: Confidence interval
CT: Computed tomography
FP: False positive
FPPI: False positive per image
FN: False negative
FROC: Free-response receiver operating characteristic
GT: Ground truth(ing)
IoU: Intersection over union
LN: Lung nodule
R1: First reader
R2: Second reader
TP: True positive

## References

[1] All Cancer Fact Sheet, World Health Organization International Agency for Research on Cancer (2020) Available via https://gco.iarc.fr/today/data/factsheets/cancers/39-All-cancers-fact-sheet.pdf. Accessed 18 Apr 2023

[2] Gould MK, Tang T, Liu ILA et al (2015) Recent trends in the identification of incidental pulmonary nodules. Am J Respir Crit Care Med 192:1208–1214. 10.1164/rccm.201505-0990OC 26214244 10.1164/rccm.201505-0990OC

[3] Hendrix W, Rutten M, Hendrix N et al (2023) Trends in the incidence of pulmonary nodules in chest computed tomography: 10-year results from two Dutch hospitals. Eur Radiol 33:8279–8288. 10.1007/s00330-023-09826-3 37338552 10.1007/s00330-023-09826-3 PMC10598118

[4] Lancaster HL, Heuvelmans MA, Pelgrim GJ et al (2021) Seasonal prevalence and characteristics of low-dose CT detected lung nodules in a general Dutch population. Sci Rep 11:9139. 10.1038/s41598-021-88328-y 33911102 10.1038/s41598-021-88328-y PMC8080793

[5] Do KH, Beck KS, Lee JM (2023) The growing problem of radiologist shortages: Korean perspective. Korean J Radiol 24:1173. 10.3348/kjr.2023.1010 38016676 10.3348/kjr.2023.1010 PMC10700998

[6] Martini K, Barth BK, Nguyen-Kim TDL, Baumueller S, Alkadhi H, Frauenfelder T (2016) Evaluation of pulmonary nodules and infection on chest CT with radiation dose equivalent to chest radiography: Prospective intra-individual comparison study to standard dose CT. Eur J Radiol 85:360–365. 10.1016/j.ejrad.2015.11.036 26781141 10.1016/j.ejrad.2015.11.036

[7] Cui X, Zheng S, Heuvelmans MA et al (2022) Performance of a deep learning-based lung nodule detection system as an alternative reader in a Chinese lung cancer screening program. Eur J Radiol 146:110068. 10.1016/j.ejrad.2021.110068 34871936 10.1016/j.ejrad.2021.110068

[8] Lopez Torres E, Fiorina E, Pennazio F et al (2015) Large scale validation of the M5L lung CAD on heterogeneous CT datasets. Med Phys 42:1477–1489. 10.1118/1.4907970 25832038 10.1118/1.4907970 PMC5148101

[9] Liu Z, Li L, Li T, Luo D, Wang X, Luo D (2020) Does a deep learning-based computer-assisted diagnosis system outperform conventional double reading by radiologists in distinguishing benign and malignant lung nodules? Front Oncol. 10.3389/fonc.2020.545862 10.3389/fonc.2020.545862 PMC7581733 33163395

[10] Li L, Liu Z, Huang H, Lin M, Luo D (2019) Evaluating the performance of a deep learning-based computer-aided diagnosis (DL-CAD) system for detecting and characterizing lung nodules: comparison with the performance of double reading by radiologists. Thorac Cancer 10:183–192. 10.1111/1759-7714.12931 30536611 10.1111/1759-7714.12931 PMC6360226

[11] Kim DW, Jang HY, Kim KW, Shin Y, Park SH (2019) Design characteristics of studies reporting the performance of artificial intelligence algorithms for diagnostic analysis of medical images: results from recently published papers. Korean J Radiol 20:405. 10.3348/kjr.2019.0025 30799571 10.3348/kjr.2019.0025 PMC6389801

[12] Yu AC, Mohajer B, Eng J (2022) External validation of deep learning algorithms for radiologic diagnosis: a systematic review. Radiol Artif Intell. 10.1148/ryai.210064 10.1148/ryai.210064 PMC9152694 35652114

[13] Winkler Wille MM, van Riel SJ, Saghir Z et al (2015) Predictive accuracy of the PanCan lung cancer risk prediction model -external validation based on CT from the Danish lung cancer screening trial. Eur Radiol 25:3093–3099. 10.1007/s00330-015-3689-0 25764091 10.1007/s00330-015-3689-0

[14] Callister MEJ, Baldwin DR, Akram AR et al (2015) British Thoracic Society guidelines for the investigation and management of pulmonary nodules. Thorax 70:ii1–ii54. 10.1136/thoraxjnl-2015-207168 26082159 10.1136/thoraxjnl-2015-207168

[15] MacMahon H, Naidich DP, Goo JM et al (2017) Guidelines for management of incidental pulmonary nodules detected on CT images: from the Fleischner Society 2017. Radiology 284:228–243. 10.1148/radiol.2017161659 28240562 10.1148/radiol.2017161659

[16] Chelala L, Hossain R, Kazerooni EA, Christensen JD, Dyer DS, White CS (2021) Lung-RADS Version 1.1: challenges and a Look ahead, from the AJR special series on radiology reporting and data systems. AJR Am J Roentgenol 216:1411–1422. 10.2214/AJR.20.24807 33470834 10.2214/AJR.20.24807

[17] Godoy MCB, Naidich DP (2009) Subsolid pulmonary nodules and the spectrum of peripheral adenocarcinomas of the lung: recommended interim guidelines for assessment and management. Radiology 253:606–622. 10.1148/radiol.2533090179 19952025 10.1148/radiol.2533090179

[18] Austin JH, Müller NL, Friedman PJ et al (1996) Glossary of terms for CT of the lungs: recommendations of the Nomenclature Committee of the Fleischner Society. Radiology 200:327–331. 10.1148/radiology.200.2.8685321 8685321 10.1148/radiology.200.2.8685321

[19] Oudkerk M, Liu S, Heuvelmans MA, Walter JE, Field JK (2021) Lung cancer LDCT screening and mortality reduction - evidence, pitfalls and future perspectives. Nat Rev Clin Oncol 18:135–151. 10.1038/s41571-020-00432-6 33046839 10.1038/s41571-020-00432-6

[20] qCT-Lung: Catching lung cancer early, qure.ai (2021) Available via https://www.qure.ai/blog/qct-lung-catching-lung-cancer-early. Accessed 13 Apr 2023

[21] Krist AH, Davidson KW, Mangione CM et al (2021) Screening for Lung Cancer: US Preventive Services Task Force Recommendation Statement. JAMA 325:962. 10.1001/jama.2021.1117 33687470 10.1001/jama.2021.1117

[22] Chakraborty DP (2021) Observer Performance Methods for Diagnostic Imaging: Foundations, Modeling, and Applications with R-based Examples. CRC PRESS

[23] Intersection over Union (IoU) in Object Detection & Segmentation, LearnOpenCV (2022) Available via https://learnopencv.com/intersection-over-union-iou-in-object-detection-and-segmentation Accessed 20 May 2023

[24] Chakraborty D (2017) The RJafroc Book: Analyzing Diagnostic Observer Performance Studies. CRC Press. Available via: https://github.com/dpc10ster/RJafrocBook/blob/gh-pages/RJafrocBook.pdf. Accessed 12 May 2023

[25] Zhou XH, Obuchowski NA, McClish DK (2011) Statistical methods in diagnostic medicine. Wiley series in probability and statistics. 10.1002/9780470906514

[26] Lo SB, Freedman MT, Gillis LB, White CS, Mun SK (2018) JOURNAL CLUB: computer-aided detection of lung nodules on CT with a computerized pulmonary vessel suppressed function. AJR Am J Roentgenol 210:480–488. 10.2214/AJR.17.18718 29336601 10.2214/AJR.17.18718

[27] Murchison JT, Ritchie G, Senyszak D et al (2022) Validation of a deep learning computer aided system for CT based lung nodule detection, classification, and growth rate estimation in a routine clinical population. PLoS One 17:e0266799. 10.1371/journal.pone.0266799 10.1371/journal.pone.0266799 PMC9070877 35511758

[28] Ming S, Yang W, Cui SJ, Huang S, Gong XY (2019) Consistency of radiologists in identifying pulmonary nodules based on low-dose computed tomography. J Thorac Dis 11:2973–2980. 10.21037/jtd.2019.07.52 31463127 10.21037/jtd.2019.07.52 PMC6687997

[29] Chen H, Huang H, Zhang J et al (2022) Intra- and inter-reader variations in lung nodule measurements: influences of nodule size, location, and observers. Diagnostics (Basel) 12:2319. 10.3390/diagnostics12102319 36292008 10.3390/diagnostics12102319 PMC9600531

[30] Azour L, Moore WH, O'Donnell T et al (2022) Inter-reader variability of volumetric subsolid pulmonary nodule radiomic features. Acad Radiol 29:S98–S107. 10.1016/j.acra.2021.01.026 33610452 10.1016/j.acra.2021.01.026

[31] Obuchowski NA, Remer EM, Sakaie K et al (2021) Importance of incorporating quantitative imaging biomarker technical performance characteristics when estimating treatment effects. Clin Trials 18:197–206. 10.1177/1740774520981934 33426918 10.1177/1740774520981934

[32] Revel MP, Bissery A, Bienvenu M, Aycard L, Lefort C, Frija G (2004) Are two-dimensional CT measurements of small noncalcified pulmonary nodules reliable? Radiology 231:453–458. 10.1148/radiol.2312030167 15128990 10.1148/radiol.2312030167

[33] Eisenhauer EA, Therasse P, Bogaerts J et al (2009) New response evaluation criteria in solid tumours: revised RECIST guideline (version 1.1). Eur J Cancer 45:228–247. 10.1016/j.ejca.2008.10.026 19097774 10.1016/j.ejca.2008.10.026

[34] Xujiong Ye YE, Xinyu Lin L, Dehmeshki J, Slabaugh G, Beddoe G (2009) Shape-based computer-aided detection of lung nodules in thoracic CT images. IEEE Trans bio-medical Engineer 56:1810–1820. 10.1109/TBME.2009.2017027 10.1109/TBME.2009.2017027 19527950

[35] Mazzone PJ, Lam L (2022) Evaluating the patient with a pulmonary nodule: a review. JAMA 327:264. 10.1001/jama.2021.24287 35040882 10.1001/jama.2021.24287

[36] Boverhof BJ, Redekop WK, Bos D et al (2024) Radiology AI Deployment and Assessment Rubric (RADAR) to bring value-based AI into radiological practice. Insights Imaging 15:34. 10.1186/s13244-023-01599-z 38315288 10.1186/s13244-023-01599-z PMC10844175

