# Supplementary material for: Validation of a commercially available CAD-system for lung nodule detection and characterization using CT-scans
Source: Eur Radiol. 2024 Jul 23;35(2):1076–88. doi: 10.1007/s00330-024-10969-0 (PMC11782423; doi:10.1007/s00330-024-10969-0)
Supplement: Supplementary file 1 — Supplementary Material [file 330_2024_10969_MOESM1_ESM.pdf]

Validation of a commercially available CAD-system for lung nodule detection and characterization using CT-scans

ELECTRONIC SUPPLEMENTARY MATERIAL

Sample size
For sample size calculation, we considered the possibility of multiple nodules being present within the same scan (correlated data- no simple random sampling). The NELSON study assessed the effectiveness of CT screening for lung cancer in high-risk individuals (smokers aged 55> years). In the NELSON trial, nodule-containing scans had an average of approximately 2 nodules[I]. Since the population planned to be included in our study are not necessarily high-risk subjects, the average number was assumed to be between 1.3 and 1.6 per scan. This estimate is based on literature stating that approximately 50% of all CT scans reveal the presence of one or more lung nodules[II]. Furthermore, around 40% of these scans with detected nodules contain more than two nodules per scan[III]. Additionally, our dataset, sourced from a cohort at a University Medical Center, is anticipated to have a higher proportion of patients over 55 years of age, a demographic associated with an increased risk of lung lesions.

Assuming a conservative within-subject correlation of 0.5, and based on internal validation results showing a nodule-level sensitivity of 80-85%, we aimed for a precision of 10%. With a type I error of 5% (two-sided) and 80% power[IV], our study required approximately 110 nodule-containing scans, alongside a similar number of non-nodule-containing scans.

Abbreviations:
NELSON - Dutch Belgian randomised lung cancer screening trial

References:
[I] Heuvelmans MA, Walter JE, Peters RB et al (2017) Relationship between nodule count and lung cancer probability in baseline CT lung cancer screening: The NELSON study. Lung Cancer. DOI: 10.1016/j.lungcan.2017.08.023

[II] Hendrix W, Rutten M, Hendrix N, et al (2023) Trends in the incidence of pulmonary nodules in chest computed tomography: 10-year results from two Dutch hospitals. Eur Radiol. DOI:10.1007/s00330-023-09826-3

[III] Cai J, Vonder M, Heuvelmans MA, et al (2022) CT characteristics of solid pulmonary nodules of never smokers versus smokers: A population-based study. Eur J Radiol. DOI:10.1016/j.ejrad.2022.110410

[IV] Zhou XH, Obuchowski NA, McClish DK (2011) Statistical methods in diagnostic medicine. Wiley series in probability and statistics. DOI:10.1002/9780470906514

Table S1 Case Stratification:

Contrast enhanced	Texture nodule	Minimal number of cases
-	Solid	20
-	Non-solid	20
-	Part-solid	20
-	No nodules	60
+	Solid	20
+	Non-solid	20
+	Part-solid	20
+	No nodules	60

Specifications CAD

Qure.ai's qCTv1.1 software is powered by a convolutional neural network (CNN), which can be used to interpret, detect, label, visualize, characterize, and monitor the progression of lung nodules in Chest CT scans[V][VI]. It functions as a secondary or concurrent reader, capable of processing both contrast and non-contrast CT (NCCT) scans. Additionally, qCT offers the option to calculate the Brock score for each nodule, estimating nodule size, type, lobular localization, count, and the presence of speculation (**Fig. S1**). The processing time per scan typically ranges between 3 to 5 minutes, depending on the number of slices in the scan.

The algorithm has been trained on a dataset comprising over 200,000 chest CT scans sourced from diverse regions including Europe, the United States, Asia, Africa, and South America. These scans were acquired using a range of scanner types, encompassing GE, Siemens, Philips, Toshiba, Canon, and Fujifilm systems.
Internal validation has been conducted using 3,200 CT scans[V].

A summary of qCT's performance metrics is provided in **Table S2**.

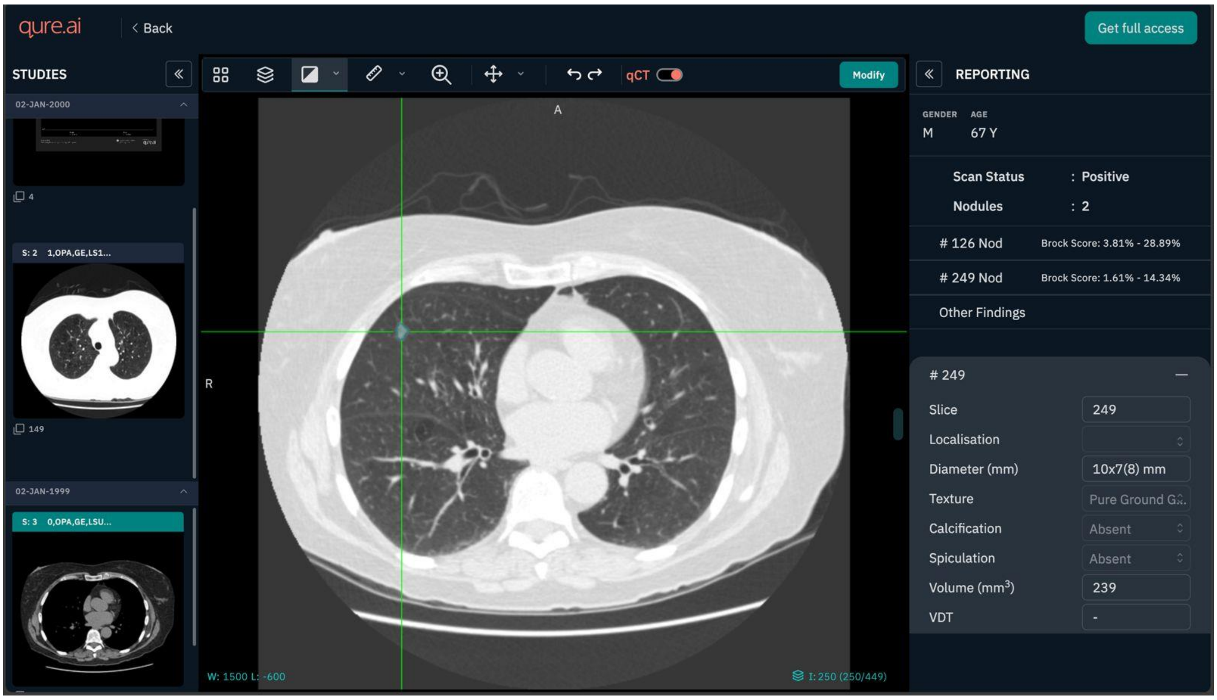

Fig. S1: qCT user interface.

Table S2 Internal Performance Metrics:

	Sensitivity	Specificity	AUC (95% CI)	Dice
**Nodule detection**	0.82 (at one False Positive Per scan)			
**Calcification**	0.91 ± 0.02	0.95	0.96 (0.95 - 0.96)	
**Spiculation**	0.82 ± 0.01	0.82	0.88 (0.87 - 0.88)	
**Texture**	0.82 ± 0.02	0.9	0.78 (0.77 - 0.79)	
**Nodule Segmentation**				0.82 (0.79-0.85)
**Lung Segmentation**				0.99(0.985-0.996)
**Lobe Segmentation**				0.96 (0.95-0.96)

References:
[V] Qure.ai Technologies Pvt Ltd (2023) qCT Instructions for Use. (Availability: Not Publicly Available)

[VI] qCT-Lung: Catching lung cancer early, qure.ai (2021) Available via https://www.qure.ai/blog/qct-lung-catching-lung-cancer-early. Accessed 13 Apr 2023

Statistical analysis packages

The statistical analyses were performed using R version 4.1.2 (R Core Team, 2021) within the RStudio environment, version 2022.12.0+353 (RStudio Team, 2021). Data wrangling was carried out using a combination of tidyverse and base R functions. Furthermore, the DescTools package was utilized to calculate the 95% confidence intervals for proportions, while the ROCit package was employed to compute the Area Under the Received Operating Characterization Curve (AUC-ROC). For generating Precision-Recall (PR) curves, the PRROC package was utilized. Additionally, the Bland–Altman plot was generated using the blandr package in R.
